# QuickStats: Homicide* and Suicide^†^ Death Rates^§^ for Persons Aged 15–19 Years — National Vital Statistics System, United States, 1999–2016

**DOI:** 10.15585/mmwr.mm6722a7

**Published:** 2018-06-08

**Figure:**
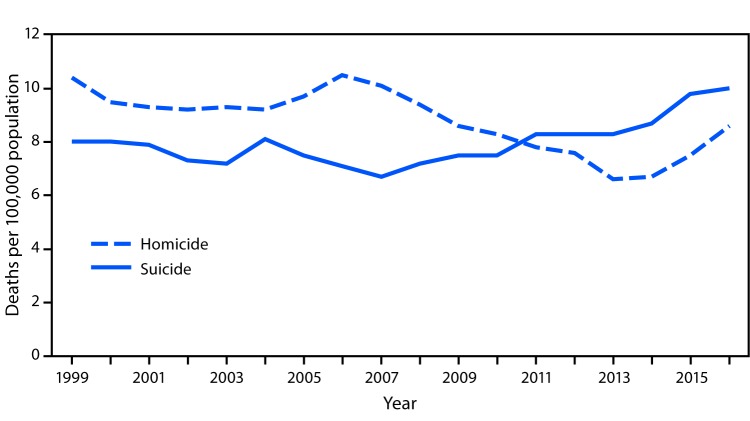
In 1999, the homicide death rate for persons aged 15–19 years (10.4 per 100,000) was higher than the suicide rate (8.0). By 2010–2011, the homicide and suicide rates had converged. After 2011, the suicide rate increased to 10.0 in 2016; the homicide rate declined through 2013 but then increased to 8.6 in 2016.

**Source: **National Vital Statistics System. 1999–2016. https://www.cdc.gov/nchs/nvss/deaths.htm

**Reported by: **Sally C. Curtin, MA, scurtin@cdc.gov, 301-458-4142; Arialdi M. Minino, MPH.

For more information on this topic, CDC recommends the following link: https://www.cdc.gov/violenceprevention/index.html.

